# Optimization Design and SLM Manufacturing of Porous Titanium Alloy Femoral Stem

**DOI:** 10.3390/ma17194896

**Published:** 2024-10-06

**Authors:** Lisong Zhao, Yukang Wang, Qing Wang, Yongdi Zhang, Guang Yang

**Affiliations:** 1 College of Mechanical Engineering, Hebei University of Science and Technology, Shijiazhuang 050018, China; 2 Hebei Provincial Collaborative Innovation Center for General Aviation Additive Manufacturing, Shijiazhuang 050018, China

**Keywords:** porous titanium alloy, topology optimization, mechanical properties, permeability, femoral stem, selective laser melting

## Abstract

In order to solve the loosening problem caused by stress shielding of femoral stem prostheses in clinical practice, an optimization design method of a personalized porous titanium alloy femoral stem is proposed. According to the stress characteristics of the femur, the porous unit cell structures (TO-C, TO-T, TO-B) under three different loads of compression, torsion, and bending were designed by topology optimization. The mechanical properties and permeability of different structures were studied. Combined with the porous structure optimization, a personalized radial gradient porous titanium alloy femoral stem was designed and manufactured by selective laser melting (SLM) technology. The results show that the TO-B structure has the best comprehensive performance among the three topologically optimized porous types, which is suitable for the porous filling structure of the femoral stem, and the SLM-formed porous femoral stem has good quality. The feasibility of the personalized design and manufacture of porous titanium alloy implants is verified, which can provide a theoretical basis for the optimal design of implants in different parts.

## 1. Introduction

In recent years, more and more people have suffered from hip joint diseases, resulting in a surge in the number of patients requiring femoral stem replacement every year. In walking and other daily activities, the trabecular bone of the femur often bears high-intensity loads such as compression, bending, and torsion, so maintaining its different types of mechanical properties is crucial to the formation of bone tissue [1]. Natural bone has two basic structures: cortical bone and cancellous bone [2]. The porosity of the outer cortical bone (~10%) is low, which provides sufficient mechanical strength to withstand human weight and different movements. The porosity of the inner cancellous bone is about 50~90%, which provides a large circulation space for the transport of nutrients and the discharge of metabolic waste [3,4].

As a commonly used orthopedic implant material, Ti6Al4V has an elastic modulus of about 110 GPa, but the elastic modulus of human bone is 0.02–20 GPa [5,6]. The elastic modulus of dense titanium alloy is still high compared with it. After implantation, it will produce a ‘stress shielding’ effect, which directly leads to the reduction of implant life or surgical failure and cannot meet the needs of personalized design. The porous structure can reduce the elastic modulus, which is widely considered an effective method to solve the mismatch of elastic modulus [7]. The porous structure is also conducive to the adhesion, proliferation, and differentiation of osteoblasts. The interconnected pores can enable the free transport of body fluids, take away metabolic waste, and promote the growth of new bone tissue into the pores. Selective Laser Melting (SLM) has high manufacturing precision, which can realize the controllable structure, shape, and performance of porous prostheses. The mechanical properties and corrosion properties of manufactured parts can meet the requirements of personalized manufacturing of medical products [8].

Many scholars have studied the design of the porous femoral stem. Acosta-Sánchez et al. [9] proposed a femoral stem prosthesis with porous structure to reduce stiffness and allow bone growth and more effective load transfer. Alkhatib et al. [10] applied the BCC structure to the design of the proximal region of the femoral stem and obtained a better effect in terms of reducing stress shielding. Jetté et al. [11] and Mehboob et al. [12] applied the porous structure to some areas of the femoral stem and found that the femoral stem designed by this method can effectively reduce the stress shielding effect. Xue et al. [13] combined density-based topology optimization with porous structure to design a new type of sleeve and femoral stem composite prosthesis and used finite element analysis to evaluate the biomechanical changes of the optimized sleeve. The results showed that the designed composite sleeve and prosthesis stem could effectively improve the biomechanical properties of the next generation of prostheses and provide a microenvironment for bone inward growth.

In summary, although there have been research and design cases of porous femoral stems, they are mainly porous in the proximal part, and the distal dense metal will affect the ingrowth of bone tissue, which is not conducive to long-term stability after implantation. Moreover, the porous structure used is not designed according to the stress condition of the human body, and there is no comprehensive evaluation of the mechanical properties and permeability under different loads. Therefore, it is necessary to design the porous structure according to the stress characteristics of the femur, realize the optimal design of the femoral implant under the premise of satisfying the mechanical properties and permeability, and explore the design and manufacturing method of the fully porous titanium alloy femoral stem, which is consistent with the radial gradient distribution characteristics of human trabecular bone, so as to reduce the stress shielding phenomenon after implantation and improve its service life in the human body.

## 2. Optimization Design of Porous Structure Based on the Stress Characteristics of Femur

According to the stress characteristics of the femur, the compression, torsion, and bending loads are applied to the 1.5 mm × 1.5 mm × 1.5 mm square solid block in the finite element analysis software ANSYS 2019 R2 (ANSYS Inc., Canonsburg, PA, USA) and the porous unit cell structure that can withstand different loads is obtained by topology optimization. In order to ensure the continuity and periodicity of the obtained model, the hexahedral mesh is used for meshing. The mesh size is 0.05 mm, the aspect ratio is 1, and the element mass is 0.9. The periodic symmetrical boundary conditions are defined for the cube solid element model, that is, the three planes composed of the X axis, Y axis, and Z axis are symmetrically constrained.

**Figure 1:**
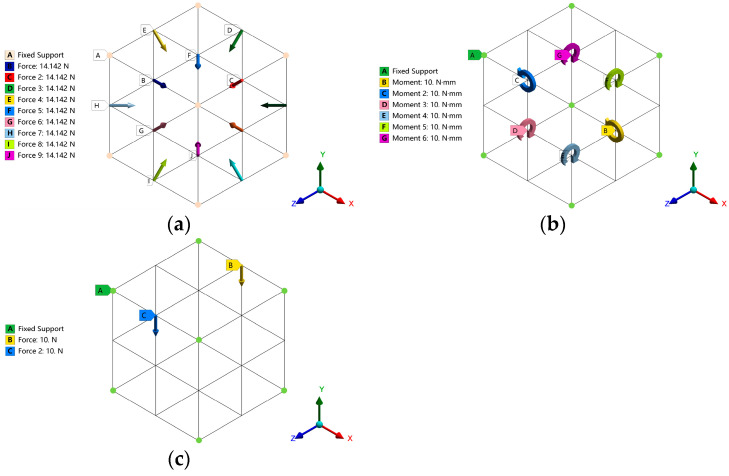
Topology optimization load boundary conditions: (**a**) compression load; (**b**) torsional load; (**c**) bending load.

The topological structure obtained by applying linear load and surface load cannot meet the circulation requirements of bone implants, and the mechanical properties cannot meet the ideal requirements. Therefore, in order to obtain a porous structure with excellent performance, this study uses point load for loading. By trying different loads, it is found that the optimization results are related to the load transfer direction and the retained volume fraction but not to the load value. Therefore, the compression, torsion, and bending load components are set to 10 N, 10 N·mm, and 10 N, respectively, and all structures retain a volume fraction of 30%. The three load boundary conditions are shown in Figure 1.

Among them, the compression load boundary condition is that all vertices are fixed, and a load component of 10 N with a size of 45° is applied downward at the midpoint of each edge. The torsional load boundary conditions are that all vertices are fixed, and a torsional load of 10 N·mm is applied at the center of each surface. The bending load boundary conditions are that all vertices are fixed, and a downward 10 N load is applied to the midpoint of the two sides above the cube to simulate the bending condition.

**Figure 2:**
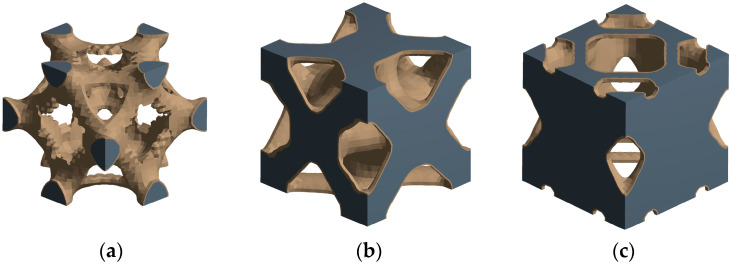
Topology optimization results: (**a**) compression; (**b**) torsion; (**c**) bending.

After the topology optimization is completed, the optimization model is output, and the results are shown in Figure 2.

**Figure 3:**
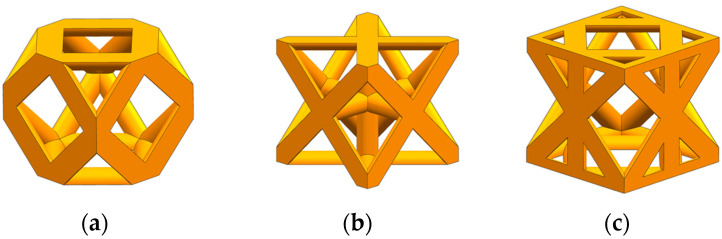
Reconstruction results of porous structure model: (**a**) TO-C; (**b**) TO-T; (**c**) TO-B.

Topology optimization can only obtain a rough structure profile, and its result file cannot be directly used for the subsequent analysis and measurement of key parameters such as aperture. It is also necessary to use three-dimensional modeling software to reconstruct the obtained topology optimization model to avoid redundant parts and surface defects. For this reason, the UG 12.0 (Siemens PLM Software, Plano, TX, USA) three-dimensional modeling software was used to reconstruct the porous unit cell after topology optimization under different loads according to the porous contour shape, and the size was 1.5 mm × 1.5 mm × 1.5 mm. According to the different loading conditions, the three structures are named TO-C (Topology-Compression), TO-T (Topology-Torsion), and TO-B (Topology-Bend). The three types of topology optimization porous unit cell models are shown in Figure 3.

**Figure 4:**
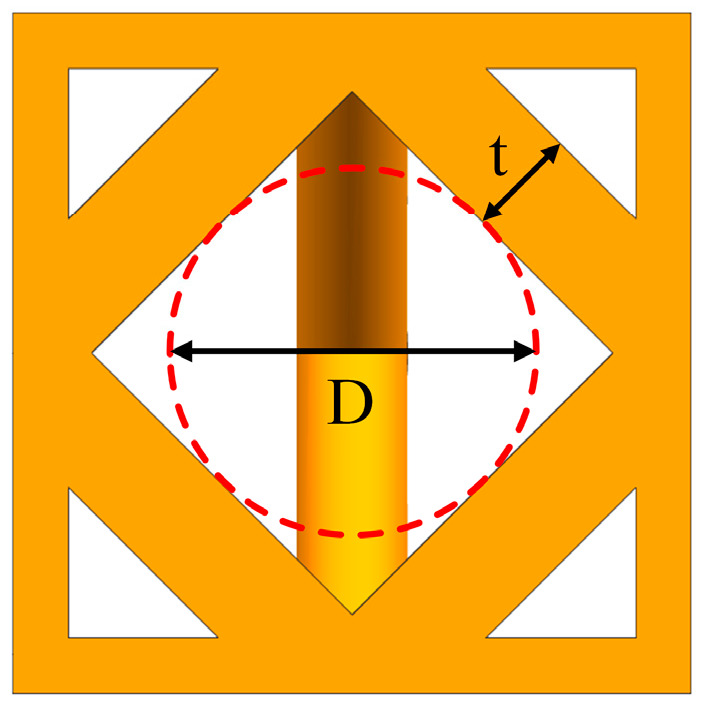
Geometric parameter diagram of porous structure.

According to the existing research, the pore size range is 300~900 μm [14,15], and the porosity ranges from 60% to 90%, factors which are helpful for the growth of bone tissues [16]. Therefore, in this study, the pore size and porosity were adjusted by changing the diameter of the strut to ensure that the parameters were within this range. The aperture is represented by the maximum inscribed circle diameter in the hole of the porous structure [17]. The diameter of the strut and the aperture are shown in Figure 4, where t represents the strut diameter, and D represents the aperture of the porous structure.

The porosity of porous structure is calculated by Equation (1) [18]:

(1) $$p=\left(1-\frac{V_{p}}{V_{s}}\right)\times 100\%$$

In the equation, *p* represents the porosity, *V_p_* is the volume of the unit cell, and *V_s_* is the volume of the compact cube unit cell.

The strut diameter–pore diameter values of the three structures are shown in Table 1. The pore diameters between 60% and 90% porosity of all structures are in the range of 600~900 μm, which can meet the conditions for human bone tissue growth.

## 3. Performances Analysis of Porous Structure

### 3.1. Subsection

According to the stress characteristics of the femur, the mechanical properties of the three porous structures were simulated in ANSYS Workbench 19.2 R2 (ANSYS Inc., Canonsburg, PA, USA). The material properties of Ti6Al4V ELI are shown in Table 2.

According to Peng et al. [19], the porous structure formed by a 2 × 2 × 2 unit cell array in compression simulation can improve the computational efficiency under the premise of ensuring the accuracy of simulation results. Therefore, in this study, the compression simulation model is set to a porous structure composed of eight units of 2 × 2 × 2.

**Figure 5:**
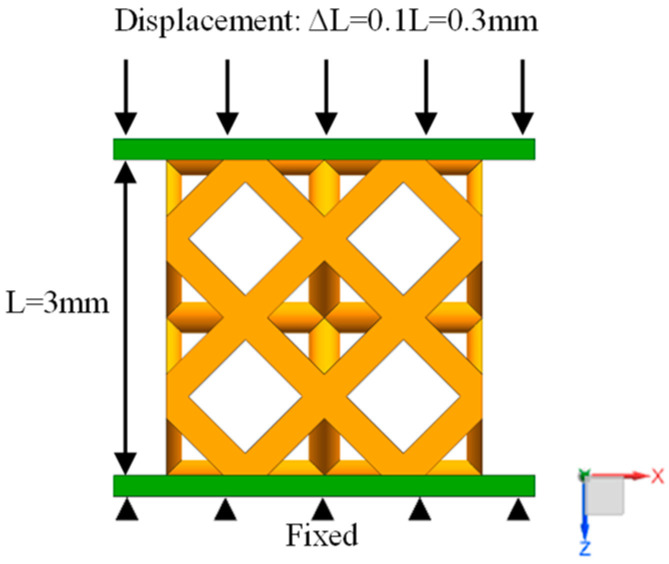
Compression simulation boundary conditions.

In order to more realistically simulate the working conditions of the indenter during compression, a rigid plate is set up above and below the model, and a friction coefficient of 0.2 is set between the porous structure and the rigid plate. The SOLID 186 tetrahedral mesh is used to mesh the model. The mesh size is 0.06 mm, which ensures that the average mesh quality of all structures is above 85%. The lower rigid plate is added with a fixed constraint, and the upper rigid plate is applied with a displacement load of 10% along the positive direction of the Z axis. The degrees of freedom of the X and Y axes are not constrained, and the compression simulation boundary conditions are shown in Figure 5.

**Figure 6:**
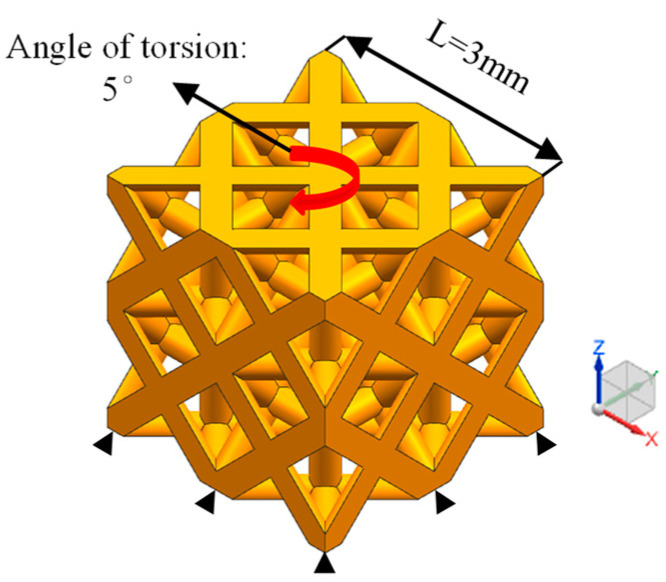
Torsion simulation boundary conditions.

Because setting the porous structure to a cylindrical shape will affect the integrity of the overall structure, the calculation accuracy of the torsion simulation results will be affected. Therefore, in this study, a square structure composed of eight units of 2 × 2 × 2 was used for torsion simulation. The mesh size division is the same as the compression simulation setting. The lower plane is fixed, and a 5° torsion angle is applied on the upper plane around the Z axis. The torsion simulation boundary conditions are shown in Figure 6.

**Figure 7:**
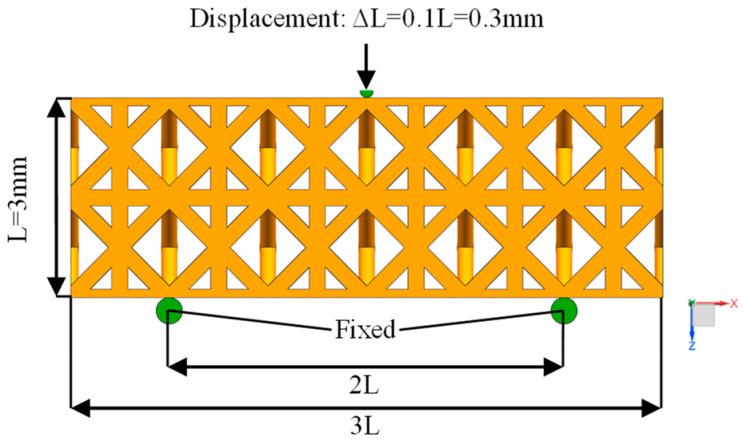
Bending simulation boundary conditions.

The three-point bending simulation of the three structures is carried out. The model is composed of a 6 × 2 × 2 unit cell array with a size of 9 mm × 3 mm × 3 mm. A semi-circular shaft is set at the center of the top of the model to simulate the indenter of the testing machine. Two fixed circular shafts are set at the bottom of the model to support the porous structure, and the support span is 6 mm. The grid size and friction coefficient settings are the same as those of the compression simulation. A displacement load of 0.3 mm is applied to the semi-circular shaft at the top center, and the three-point bending simulation boundary conditions are shown in Figure 7.

Porous orthopedic implants not only need to meet the requirements of human natural bone mechanical safety but their permeability (specific surface area, permeability coefficient) also has an important impact on cell proliferation and differentiation. Permeability affects the mass transport of nutrients, allowing cells to differentiate and proliferate better within the implant. Therefore, after studying the mechanical properties of the topologically optimized porous structure under different load conditions, the permeability of the porous structure was evaluated.

### 3.2. Preparation of Porous Structure Compression Specimens

The compression specimens were prepared by an AM 250 metal printer (Renishaw, Landon, UK). The material used was Ti6Al4V ELI titanium alloy spherical powder for metal 3D printing. The chemical composition is shown in Table 3.

**Figure 8:**
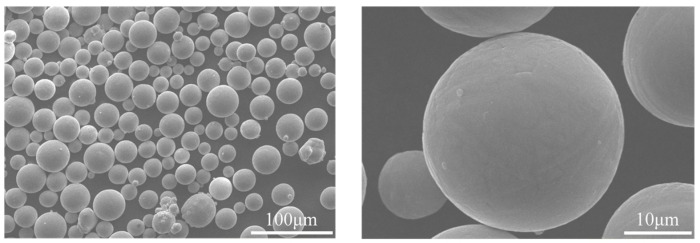
Microstructure of Ti6Al4V ELI powder.

**Figure 9:**
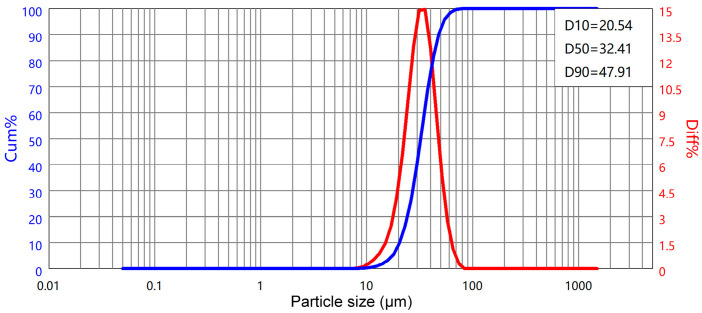
Ti6Al4V ELI powder particle size.

The powder was observed by S-4800 scanning electron microscopy (Hitachi, Tokyo, Japan). The microscopic morphology is shown in Figure 8, and it can be seen that the powder has good sphericity. Figure 9 is the powder particle size distribution map. The powder particle size range is about 20~48 μm, of which D10 = 20.54 μm, D50 = 32.41 μm, and D90 = 47.91 μm, and the Hall flow rate is 32.6 s/50 g. The powder has good fluidity to ensure a good powder-spreading effect.

In order to ensure the integrity of the specimen structure, in this study, the aspect ratio of the porous compression specimen was set to 1, and the cube compression specimen with a size of 15 mm × 15 mm × 15 mm was made of 10 × 10 × 10 cell array. The compression specimen was prepared by SLM technology, and the process parameters are shown in Table 4.

### 3.3. Permeability Analysis

The specific surface area is a description of the internal pore space of porous implants, also known as the relative surface area or surface volume ratio. In general, when the specific surface area is larger, the larger the internal pores of the porous structure, the larger the contact area, the greater the adhesion space for bone cells, and the more conducive to cell adhesion and proliferation. The specific surface area calculation method is calculated by Equation (2) [20]:

(2) $$s=\frac{S}{V}$$

In the equation, *s*, *S*, and *V* represent the specific surface area, internal surface area, and volume of the porous structure, respectively.

**Figure 10:**
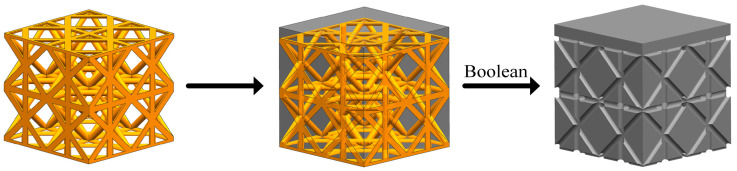
Fluid domain model establishment.

Before the fluid dynamics simulation, it is necessary to establish a fluid domain model of porous structure. Firstly, a porous structure model is constructed by using a unit cell 2 × 2 × 2 array in UG 12.0 software (Siemens PLM Software, Munich, Germany), and then a cuboid solid model is constructed on this basis. In order to simulate the real fluid flow, a 0.3 mm fluid inlet is set aside above, and the fluid domain simulation model is finally obtained through the Boolean subtraction command, as shown in Figure 10.

**Figure 11:**
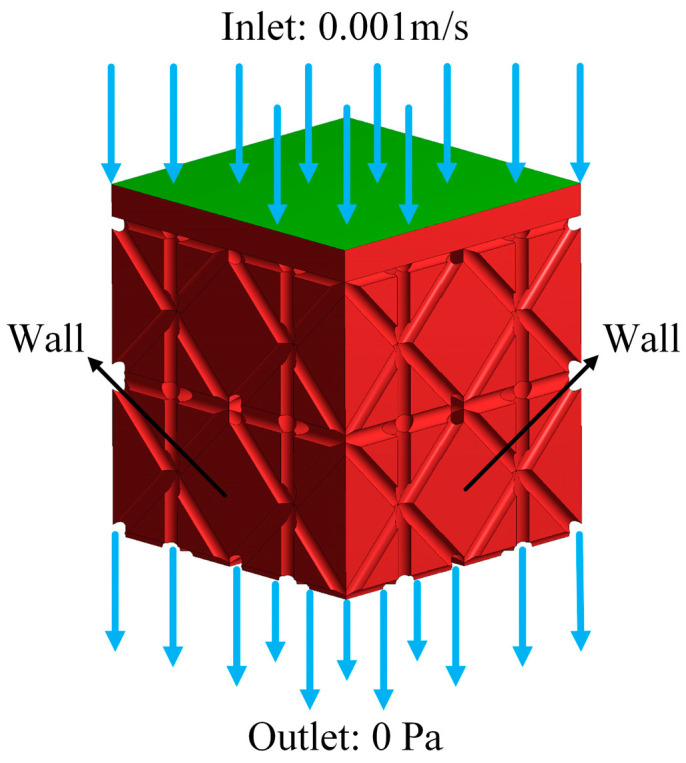
Fluid dynamics simulation boundary conditions.

In this paper, the fluid in the simulation process uses a body fluid with a density of *ρ* = 1060 kg/m^3^ and a dynamic viscosity of μ = 0.0032 Pa·s to simulate the flow of body fluid in porous implants more realistically [21]. The fluid flows from the top entrance at a speed of 0.001 m/s, and the outlet is set to a free boundary condition. The pressure is 0 MPa, and the boundary condition diagram is shown in Figure 11.

Permeability can intuitively evaluate the mass transfer characteristics of the fluid. Permeability represents the ability of the fluid to flow through porous structures and the ability to transport nutrients or metabolites in bone tissue. It is an important parameter for the in vivo performance of bone implants and is essential for cell adhesion and vascularization. Darcy’s law is usually used to measure the permeability of laminar flow through porous media. The permeability coefficient is calculated by Equation (3).

(3) $$k=\frac{Q\mu L}{A\Delta P}=v\mu \frac{L}{\Delta P}$$

In the equation, *k*, *Q*, *μ*, *L*, *A*, Δ*P*, and *v* represent the permeability coefficient, volume flow rate, dynamic viscosity of human body fluid, height of the porous structure, cross-sectional area of the porous structure, pressure drop between the inlet and outlet, and inlet velocity of porous structure, respectively.

## 4. Personalized Design of Femoral Stem

**Figure 12:**
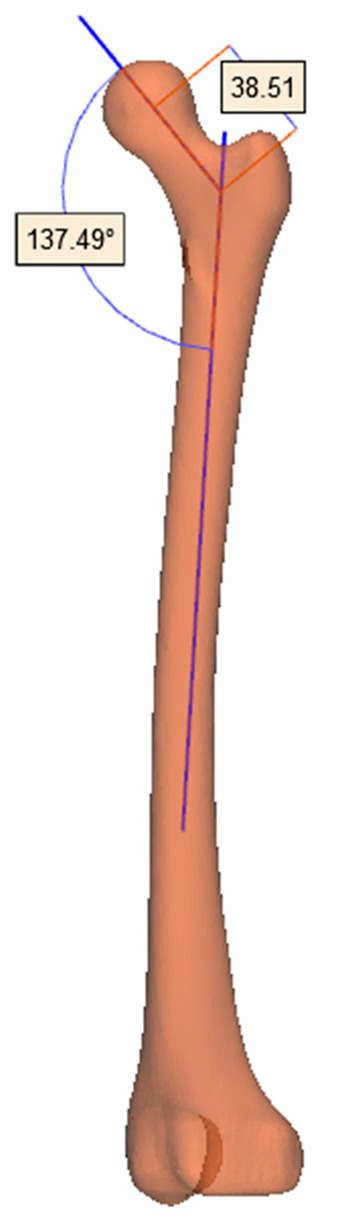
Measurement of key parameters of femur.

**Figure 13:**
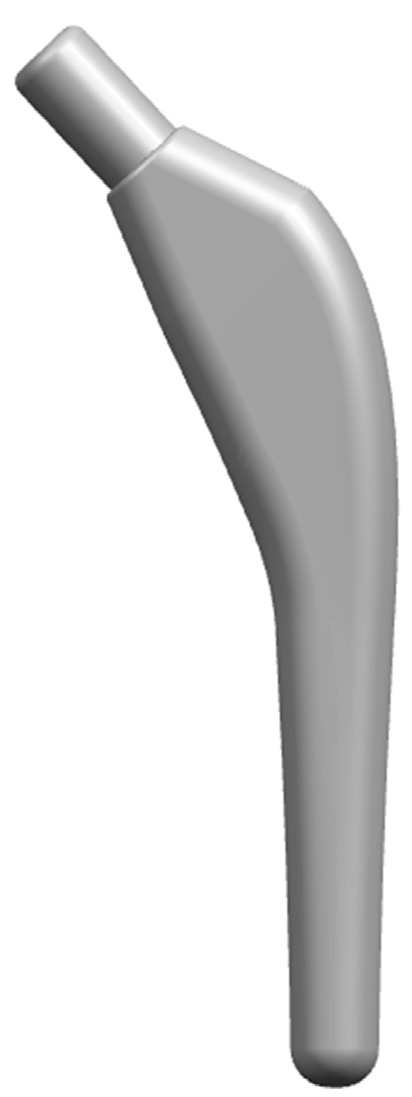
Femoral stem model.

The neck area is the weakest part of the femur. In the personalized design of the femoral stem prosthesis, the corresponding part needs to have sufficient bending strength. The key parameters of this part of the design are the neck–shaft angle and neck length, that is, it is necessary to ensure that the prosthesis angle matches the neck–shaft angle of the femur, and the top of the prosthesis should also be in the center of the femoral head. The angle between the femoral neck and the body is called the neck–shaft angle. Mimics Research 21.0 software (Materialise, Leuven, Belgium) can fit the center line of the three-dimensional model, and the data of the femoral neck–shaft angle and osteotomy angle can be measured by this method. Therefore, the femur model extracted from Mimics software (Materialise, Leuven, Belgium) is saved as an STL format file and imported into 3-Matic 12.0 software (Materialise, Leuven, Belgium) to fit the center line of the femur and femoral neck. The measured neck–shaft angle and neck length parameters are shown in Figure 12, which are 137.49° and 38.51 mm, respectively. The femoral stem model was established according to the key parameters, as shown in Figure 13.

**Figure 14:**
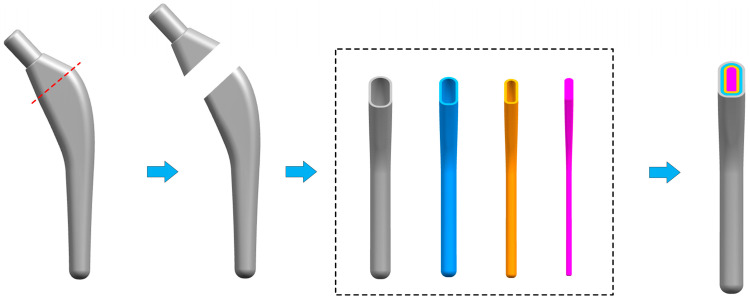
Layering diagram of femoral stem.

The dense femoral stem was cut into two parts by UG 12.0 (Siemens PLM Software, Munich, Germany), and the lower part was used for the design of the radial gradient. In order to ensure that the porous structure is completely filled into the porous layer and the struts between different porous layers can be closely connected, the solid part is stratified by the ‘shelling’ command. According to the design size of the porous unit cell, the thickness of each layer is set to 1.5 mm and finally divided into four layers. The radial stratification method and stratification results of the femoral stem are shown in Figure 14.

## 5. Results and Discussion

### 5.1. Mechanical Properties

**Figure 15:**
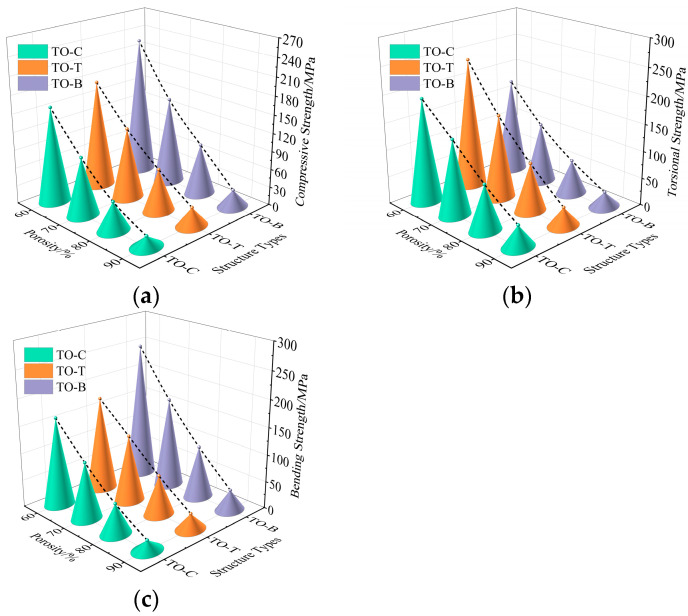
Mechanical properties simulation results: (**a**) compressive strength; (**b**) torsional strength; (**c**) bending strength.

Mechanical bearing strength is a key factor in evaluating the mechanical properties of bone implants. In this study, the mechanical properties (compressive strength, torsional strength, and bending strength) of three porous structures obtained by topology optimization were studied by finite element simulation. After obtaining the simulation results, in order to more intuitively compare the strength of the mechanical properties of each structure, a three-dimensional diagram is drawn, as shown in Figure 15.

By analyzing the simulation results of the mechanical properties of Figure 15, it can be seen that the mechanical properties of the porous structure are related to the topology type, and, with the increase in porosity, the mechanical properties show a downward trend. Because the mechanical properties of each structure are significantly different when the porosity is 60%, the mechanical properties of each structure at 60% porosity are drawn as shown in Table 5. Combined with Figure 15 and Table 5, the mechanical properties of the three structures can be clearly distinguished. The compressive strength and bending strength are TO-B > TO-T > TO-C, and the torsional strength is TO-T > TO-C > TO-B.

**Figure 16:**
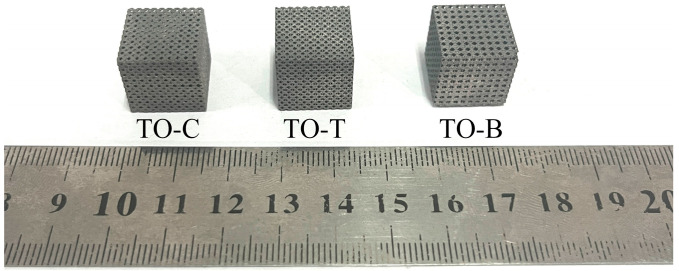
SLM forming effect of porous specimens.

The compression specimens of three porous structures at 60% porosity were manufactured by AM 250 metal printer (Renishaw, Landon, UK). Three specimens were prepared for compression tests for each structure type. After printing, the specimens were cut off by a wire-cutting machine (Huafang, Hangzhou, China). After cleaning with an ultrasonic cleaner (ChunRain, Shenzhen, China), the specimens were sandblasted (Jichuan, Dongguan, China) to ensure that they had good surface quality and to remove part of the unmelted Ti6Al4V ELI powder in the structure, avoiding adverse effects on the accuracy of the test results. The three types of specimens completed by treatment are shown in Figure 16. It can be seen that the pore distribution of the specimens is obvious, and the molding quality is good.

**Figure 17:**
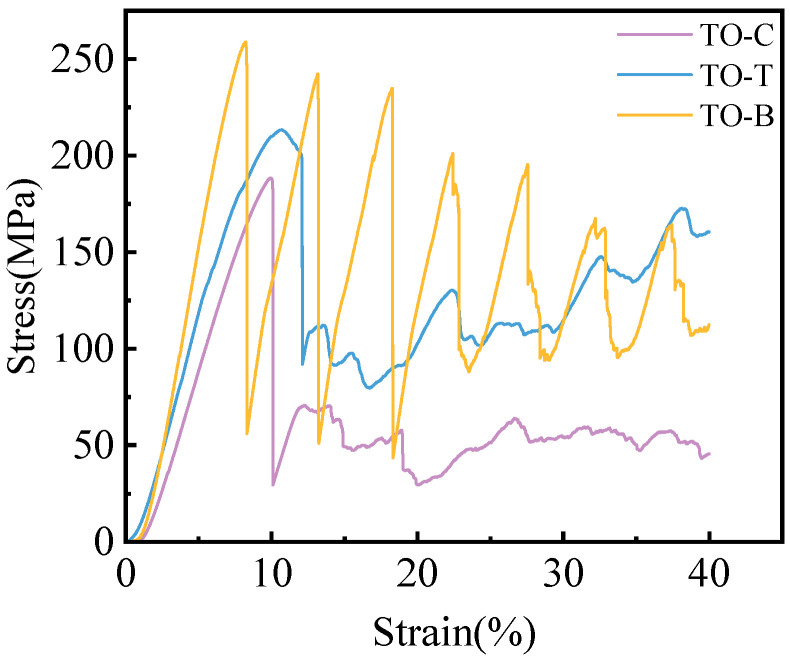
Compression stress–strain curve of porous specimen.

The compression test equipment used is the CMT 5105 micro-controlled electronic universal testing machine (MTS systems China Company, Shenzhen, China). Before the test, the compression rate of the testing machine was set to 1 mm/min, and the compression test was carried out on the three structural types of specimens after post-processing was carried out according to ISO 13314 [22]. After the completion of the test, the original compression data were derived, the stress and strain were calculated, and the stress–strain curve was drawn. The stress–strain curve of the 60% porosity porous specimen is shown in Figure 17.

**Figure 18:**
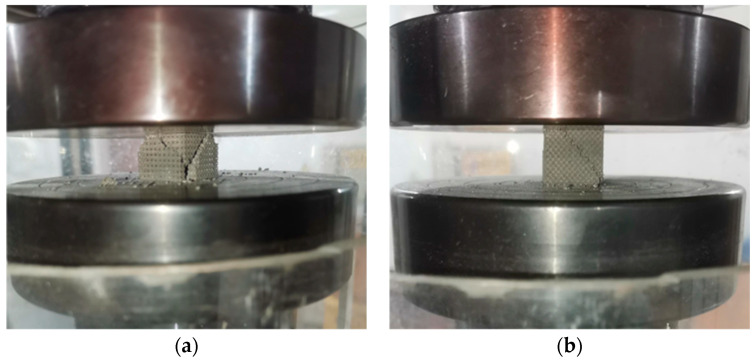
The 45° shear failure results of the porous structure: (**a**) TO-C; (**b**) TO-T.

From the stress–strain curves, the differences in the mechanical behavior of the three porous structures in compression can be analyzed, and the compression process can be divided into three main stages: the elastic stage, the sudden drop stage after the stress increase, and the final densification stage. Combined with the analysis of the compression test phenomena, it can be seen that the three structures have different failure forms in compression, as shown in Figure 18. The TO-C and TO-T structures exhibit 45° shear damage failure due to the existence of a bowed structure at the connection of their outer struts, resulting in a rapid decrease in stress and gradual leveling off after the on-line elasticity stage. The TO-C structure has a lower load-carrying capacity and a smaller peak stress, indicating its limited compressive performance. In contrast, the TO-T structure has multidirectional struts inside, which can effectively disperse the loads, and thus it has smaller fluctuations in the stress–strain curves and a significantly higher load-bearing capacity. The periodic geometric features of the TO-B structure, on the other hand, exhibit gradual yielding behaviors during compression, with each periodic cell yielding sequentially, which provides a higher energy-absorbing capacity and avoids localized stress concentration and premature failure at a single location. On the stress–strain curve, the cyclic design of the TO-B structure exhibits multiple high peak stresses and sharp upward and downward fluctuations, and this progressive yielding characteristic significantly improves its overall load-bearing and energy-absorbing capacity, demonstrating superior compressive performance.

By sorting out the compression test data, the order of compressive strength is TO-B > TO-T > TO-C, which is consistent with the trend of compression simulation results in Figure 15. The compressive strength of the TO-B structure is 258.88 MPa, which is higher than that of human cortical bone (100~230 MPa). The TO-T and TO-C structures are 213.35 MPa and 188.35 MPa, respectively, within the compressive strength range of human cortical bone, and the elastic modulus of each structure is also within the range required by human bone. The compressive strength and elastic modulus values of the three structures are shown in Table 6.

### 5.2. Permeability

The specific surface area of the porous structure in the range of 60~90% porosity is shown in Table 7.

From the data in the table, it can be concluded that the TO-B structure provides the largest specific surface area, which can contribute to cell proliferation and differentiation and promote bone tissue ingrowth.

**Figure 19:**
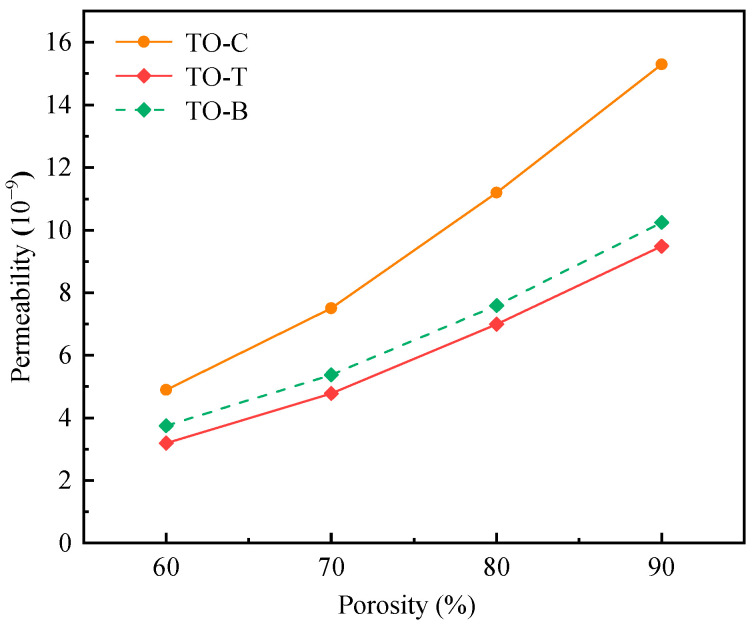
Permeability coefficient of different structures.

For porous implants, permeability can be regarded as the transport capacity of body fluids (nutrients and metabolites) inside the porous structure. Good permeability can allow more nutrients to flow inside the porous structure, thereby promoting bone tissue regeneration and improving the success rate of implantation. The relationship between the permeability and porosity of each structure is shown in Figure 19.

It can be seen from the diagram that the permeability coefficient of the TO-C structure is significantly different from that of the TO-T and TO-B structures, and the permeability coefficient of the latter two is similar. Among them, the TO-C structure has a simple structure and fewer internal struts that hinder the fluid from passing through, so the flow rate of the fluid through the aperture is faster, and the permeability coefficient is also the highest. However, too high a permeability coefficient will also be not conducive to cell adhesion and proliferation, thus affecting the bone ingrowth of the implant. It is necessary to select the appropriate permeability coefficient.

To prove the reliability of the permeability results obtained by simulation, the permeability coefficients in different literature are compared. The permeability coefficients reported in different literature are shown in Table 8.

Combined with the research data in this paper, it can be seen that the order of magnitude (10^−9^ m^2^) of the permeability coefficient of the porous structure in this paper is the same as that in the literature listed in the table. Although the permeability coefficient is not as good as the research results of Ali et al. [23] on ordinary CAD structures, it still meets the requirements of permeability (0.0268 × 10^−9^ m^2^~20 × 10^−9^ m^2^) of trabecular bone in different parts of human beings obtained by Grimm et al. [28] and Nauman et al. [29].

### 5.3. Comprehensive Characteristic Comparison

The primary consideration of medical porous titanium alloy implants is mechanical properties, among which compressive strength, torsional strength, and bending strength are the key mechanical indexes. In addition, permeability is crucial for the biocompatibility of femoral stem implants and successful fusion with bone tissue. The larger specific surface area helps to increase the contact area between the implant and the surrounding bone tissue and promotes the growth of bone cells and bone healing. A good permeability coefficient ensures that the implant can be effectively combined with bone tissue, thereby improving the overall stability of the implant.

**Figure 20:**
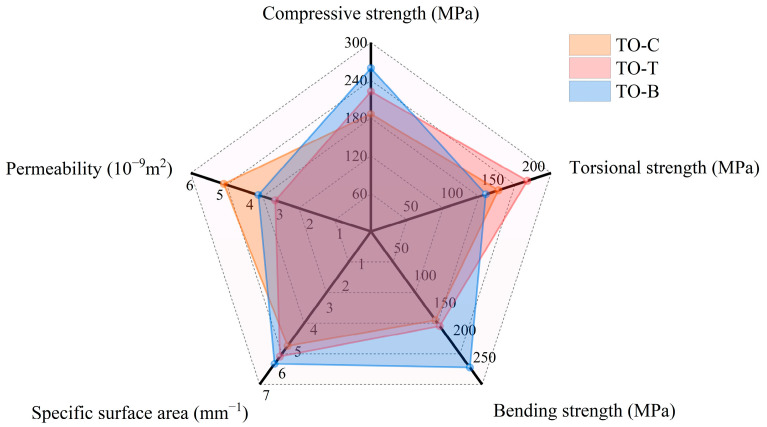
Comprehensive comparison of porous structure performance.

Therefore, in order to understand the applicability of porous titanium alloy in the field of femoral stem implantation, the properties of different porous titanium alloys designed by topology optimization are compared comprehensively, and the structure type with the best comprehensive performance is selected for the optimization design of the femoral stem. Taking the performance data of different porous structures with 60% porosity as the representative, the radar chart shown in Figure 20 is drawn to comprehensively compare the performance of porous structures.

It can be seen from the figure that TO-C has the highest permeability coefficient among the three structures, but its specific surface area and mechanical properties are at a low level. The TO-T structure has the best torsional strength, and the compressive strength and specific surface area are at a high level, but its bending strength and permeability coefficient are low; the compressive strength, bending strength, and specific surface area of the TO-B structure are optimal, and the permeability coefficient is also at a high level, but its torsional strength is the weakest. Although the permeability of the three porous structures at 60% porosity is not optimal, they can meet the needs of human bone porous implants for permeability. Therefore, the TO-B structure has the best performance among the three structures, with the best mechanical bearing capacity and better permeability (permeability coefficient and specific surface area), which is conducive to cell adhesion and bone tissue growth. Therefore, the TO-B structure is selected as the porous filling structure of the personalized femoral stem.

### 5.4. SLM Manufacturing of Porous Titanium Alloy Femoral Stem

**Figure 21:**
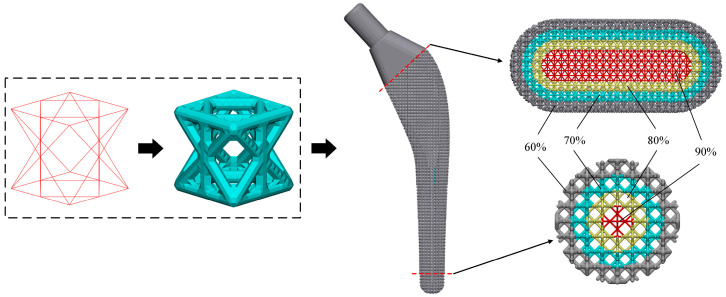
Radial gradient porous filling of femoral stem.

The porous structure TO-B with the best comprehensive performance is applied to the design optimization of the femoral stem so as to ensure that its performance meets the implantation requirements. Firstly, the TO-B unit cell structure was established from line to body in 3-Matic 12.0 software (Materialise, Leuven, Belgium), and the layered femoral handle was imported into the software, and then each layer was filled with holes. When the structure is transformed into an STL model, each layer is given different rod diameter sizes, which are 0.165 mm, 0.245 mm, 0.315 mm, and 0.385 mm from inside to outside, and the corresponding porosity is 90%, 80%, 70%, and 60%, respectively. The effect of the TO-B porous structure filling into the femoral stem is shown in Figure 21, and the distribution of the gradient porous layer can be clearly seen.

**Figure 22:**
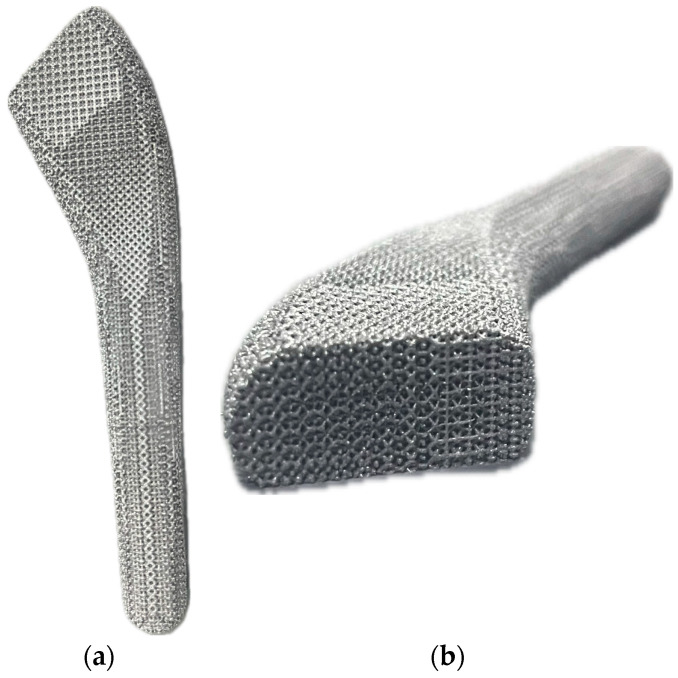
SLM forming effect of porous area of femoral stem: (**a**) front view morphology; (**b**) radial gradient morphology.

The process parameters were set as follows: laser power 200 W, scanning speed 1200 mm/s, powder layer thickness 30 μm, and scanning spacing 140 μm, and then the generated. The MTT file was imported into the system of the Renishaw equipment. Argon was filled into the molding chamber. When the oxygen content reached below 300 ppm and the substrate temperature was heated to 170 °C, the personalized porous titanium alloy femoral stem began to form. After the printing is completed, the molded part is removed by wire cutting. After ultrasonic cleaning and sandblasting, the forming effect of the final porous area is shown in Figure 22.

It can be clearly seen from the figure that the porous titanium alloy femoral stem has a good effect, the shape of the strut is clearly visible, and the strut connection between the porous filling layers is well combined, which can meet the molding quality requirements of SLM manufacturing personalized implants.

## 6. Conclusions

In this paper, according to the stress characteristics of the femur, the porous unit cell structure under different loads is designed by topology optimization. Through the study of mechanical properties and permeability of different structures, the best porous structure suitable for the femoral stem is determined. Finally, the personalized porous titanium alloy femoral stem is optimized and designed, and the SLM technology is used for forming and manufacturing. The feasibility of personalized manufacturing of porous titanium alloy implants is verified. The main conclusions are as follows:

1. According to the characteristics of compression, torsion, and bending load of the femur, three structures composed of TO-C, TO-T, and TO-B are designed by topology optimization, and the three structures are reconfigured by UG 12.0 (Siemens PLM Software, Plano, TX, USA).
2. The mechanical properties (compressive strength, torsional strength, and bending strength) of three kinds of porous structures are simulated by the finite element method. The results show that the compressive and bending properties of the TO-B structure are the best. When the porosity is 60~90%, the simulation values of compressive strength and bending strength of the TO-B structure are 28.37~228.13 MPa and 37.82~249.08 MPa, respectively. The torsional performance of the TO-T structure is the best. When the porosity is 60~90%, the simulation value of the torsional strength of the TO-T structure is 34.29~238.36 MPa. The compressive specimen with 60% porosity was prepared by SLM technology, and the compressive test was carried out. The results show that the compressive performance trend of the three structures is similar to the simulation, and the TO-B structure has the highest compressive strength and energy absorption.
3. The fluid dynamics simulation of the porous structure was carried out, and the permeability coefficients of the three structures with 60~90% porosity were between 3.19 × 10^−9^ m^2^~15.3 × 10^−9^ m^2^, which all met the permeability requirements of human trabecular bone.
4. The properties (compressive strength, torsional strength, bending strength, permeability, and specific surface area) of the three structures were comprehensively compared. By comparison, TO-B was selected as the optimum porous structure type for femoral stem porous filling. According to the characteristics of the radial gradient distribution of the internal and external density of the human trabecular bone, the femoral stem is divided into four layers in the radial direction, and the TO-B porous structure is filled into the porous layer. The porosity is 90%, 80%, 70%, and 60%, respectively, from the inside to the outside so as to realize the optimal design of the femoral stem. Finally, the personalized porous titanium alloy femoral stem was formed by SLM technology.

Although the personalized porous titanium alloy femoral stem was manufactured by SLM after design optimization, there is still some work to be studied in the design and manufacturing process.

1. Only the compression test of the porous titanium alloy directly formed by SLM was carried out. However, due to the limitation of the forming process, residual stress will inevitably occur during the forming process. It is necessary to explore the effect of heat treatment on the mechanical properties of the specimen after stress relief.
2. The finite element simulation of the compression, torsion, and bending of porous structures was carried out, but only compression experiments were carried out without torsion and bending experiments. In the future, various types of mechanical experiments can be carried out on porous structures.
3. Only the optimal design of the femoral stem and SLM printing were completed. The stiffness test or fatigue performance test of the SLM-formed porous femoral stem can be continued to verify whether the formed prosthesis can meet the service life requirements after implantation.

## Figures and Tables

**Table 1:** Strut diameter–aperture value of unit cell model.

Structure Types	Parameters	60%	70%	80%	90%
TO-C	t/mm	0.455	0.380	0.295	0.200
	D/mm	0.606	0.681	0.766	0.861
TO-T	t/mm	0.375	0.315	0.250	0.170
	D/mm	0.689	0.746	0.811	0.891
TO-B	t/mm	0.385	0.315	0.245	0.165
	D/mm	0.676	0.746	0.816	0.896

**Table 2:** Ti6Al4V ELI material properties.

Density	Young’s Modulus	Poisson’s Ratio	Yield Strength
4.5 kg/m^3^	110 GPa	0.33	970 MPa

**Table 3:** Chemical composition of Ti6Al4V ELI (*ω/%*).

H	N	O	Fe	C	V	Al	Ti
0.012	0.05	0.08	0.13	0.25	3.5	5.5	Balance

**Table 4:** SLM molding process parameters.

Laser Power	Scanning Speed	Sweep Spacing	Layer Thickness
200 W	1200 mm/s	0.14 mm	0.03 mm

**Table 5:** Mechanical properties of porous structure with 60% porosity (MPa).

Mechanical Properties	TO-C	TO-T	TO-B
Compressive strength	158.36	177.42	228.13
Torsional strength	155.85	191.08	140.45
Bending strength	162.54	173.28	249.08

**Table 6:** Compressive properties of porous specimens with 60% porosity.

Structure Types	Compressive Strength/MPa	Elastic Modulus/GPa
TO-C	188.35	2.87
TO-T	213.35	2.51
TO-B	258.88	4.16

**Table 7:** The specific surface area of porous structure (mm^−1^).

Porosity/%	TO-C	TO-T	TO-B
60	5.24	5.72	6.06
70	4.87	5.23	5.80
80	4.22	4.51	5.16
90	3.19	3.37	3.97

**Table 8:** Comparison of permeability coefficients of porous structures in different literature.

Structure Types	Porosity/%	Permeability/10^−9^ m^2^	References
General CAD	60~90	1~25	Ali et al. [23]
TPMS	50~70	1.92~5.5	Yu et al. [24]
Voronoi	40~90	0.6~21	Chao et al. [25]
Micro-CT	78~82	0.75~1.74	Baino et al. [26]
Gradient TPMS	-	5.44~14.2	Davoodi et al. [27]
Trabecular bone	-	0.4~11	Grimm et al. [28]
	-	0.0268~20	Nauman et al. [29]
TO-C	60~90	4.9~15.3	This work
TO-T		3.19~9.49	
TO-B		3.75~10.25	

## Data Availability

Data are contained within the article.
